# Risk factors for *Plasmodium falciparum *hyperparasitaemia in malarious children

**DOI:** 10.1186/1471-2334-11-268

**Published:** 2011-10-09

**Authors:** Akintunde Sowunmi, Titilope M Okuboyejo, Grace O Gbotosho, Christian T Happi

**Affiliations:** 1 Department of Pharmacology & Therapeutics and Institute for Medical Research and Training, University of Ibadan, Ibadan, Nigeria

**Keywords:** Hyperparasitaemia, children, risk factors, Nigeria

## Abstract

**Background:**

Hyperparasitaemia is a feature of childhood severe malaria but there is little information on the risk factors for hyperparasitaemia in malarious children

**Methods:**

The risk factors associated with *Plasmodium falciparum *hyperparasitaemia, defined as asexual parasitaemia > 250,000/μl, at presentation were evaluated in 3338 malarious children enrolled prospectively between 2008 and 2010 in an endemic area of southwestern Nigeria.

**Results:**

At enrolment, 97 (3%) of 3338 malarious children had hyperparasitaemia. In a multiple regression model, 3 factors were found to be independent risk factors for the presence of hyperparasitaemia at enrolment: an age ≤ 11 years (Adjusted odds ratio [AOR] = 2.85, 95% confidence interval [CI] 1.23-6.61, P = 0.014), fever (AOR = 2.02, 95% CI 1.23-3.29, P = 0.005), and enrolment after year 2008 (AOR = 0.42, 95% CI 0.24-0.73, P = 0.002). Duration of illness ≤ 3 d was associated with increased risk of hyperparasitaemia. There was no association between season and hyperparasitaemia. Compared to non-hyperparasitaemia, hyperparasitaemia was associated with an increased risk of progression to cerebral malaria (P < 0.0001). The risk of progression in hyperparasitaemic children was higher in < 5-year olds (P = 0.02).

**Conclusion:**

Young age and presence of fever are independent risk factors for hyperparasitaemia which is associated with an increased risk of progression to cerebral malaria. The findings have implications for case and community management of childhood hyperparasitaemia and for malaria control efforts in sub-Saharan Africa where severe malaria is relatively common.

## Background

*Plasmodium falciparum *hyperparasitaemia, defined as > 250,000 asexual parasites/μl blood or > 4% parasitized erythrocytes [1,2], is a feature of severe childhood malaria, is indicative of a large sequestered parasite biomass, and poses a risk for recrudescent infections following antimalarial drug treatment [3,4]. Hyperparasitaemia is relatively more frequent in the relatively non-immune individuals [1] but may also occur in semi-immune populations with much variability in clinical features [5]. However, in some non-endemic populations, for example in Indian children, it is associated with increased risk of mortality [6].

Delay in parasite clearance by all antimalarial drugs is associated with increasing parasitaemia and hyperparasitaemia [4,7] and is thought to be contributory to drug treatment failure in *P. falciparum *to antimalarials including artemisinin derivatives and artemisinin-based combinations [8]. A recent study from Thailand [9], an area of lesser intensity of malaria transmission than Nigeria [10], has shown that hyperparasitaemia is a risk factor for gametocyte carriage. Although hyperparasitaemia is frequently reported in many antimalarial and malaria studies there is little or no information on the risk factors for hyperparasitaemia in childhood falciparum infections. Such information is necessary as it may potentially harness the efforts aimed at the management and control of drug resistance in both the individual and the community. In the present study we evaluated the factors that influence the occurrence of hyperparasitaemia in children presenting with acute, symptomatic, uncomplicated, *P. falciparum *malaria in a hyperendemic area of malaria in southwest Nigeria and describe the risk of hyperparasitaemia in these children.

## Methods

### Patients

The study was carried out between January 2008 and December 2010 in patients presenting at the Malaria Clinic at the University College Hospital in Ibadan, an endemic area for malaria in southwestern Nigeria [10]. In this area, transmission occurs all year round but is more intense during the rainy season from April to October. *P. falciparum *is the predominant species accounting for 99% of all infections. Children are more affected than adults, and apparently asymptomatic infections occur in older school children and adults.

During the study period, all children with febrile illnesses suspected to be or clinically diagnosed as acute falciparum malaria were enrolled in the study after obtaining a written informed consent from parents or guardian of the children. Clinical evaluation consisted of a general clinical examination including measurement of weight, core temperature and physical examination. Core temperature was measured orally using an electronic thermometer. In very few young children it was measured rectally. Ethical clearance was provided by the local ethics committee.

### Drug treatment

Patients with parasitologically proven *P. falciparum *mono infections who were not included in 5 antimalarial efficacy trials (n = 2408) (see below) were treated with amodiaquine-artesunate and followed for approximately 2-4 weeks. Patients who met the inclusion criteria for enrolment into drug efficacy trials (n = 930) involving artesunate alone, mefoloquine alone, artesunate-mefloquine, artesunate-amodiaquine and artemether-lumefantrine were enrolled into these studies and have been reported elsewhere [11-16].

### Assessment of parasitaemia

Thick and thin blood films prepared from a finger prick were Giemsa-stained and were examined by light microscopy under an oil-immersion objective, at × 1000 magnification, by two independent assessors (microscopists). A senior member of the study team reviewed the slides if there was any disagreement between the two microscopists. In addition, one in every four blood slides was reviewed by this senior member. Parasitaemia in thick films was estimated by counting asexual parasites relative to 1000 leukocytes, or 500 asexual forms, whichever occurred first. From this figure, the parasite density was calculated assuming a leukocyte count of 6000/mL of blood. Gametocytes were also counted in thick blood films against 1000 leukocytes assuming an average leukocyte count of 6000/mL of blood [17-19]. Haematocrit was done at enrolment in 1794 children.

### Statistical analysis

Data were analysed using version 6 of the Epi-Info software [20], and the statistical program SPSS for Windows version 10.01 [21]. Proportions were compared by calculating Chi square with Yates' correction. Normally distributed, continuous data were compared by Student's t-tests and analysis of variance (ANOVA). Data not conforming to a normal distribution were compared by the Mann-Whitney U-test and the Kruskal-Wallis test (or by Wilcoxon rank sum test). The association between body temperature and parasite density was assessed by Spearman's correlation coefficient. Semi-log plot of mean parasite density versus temperature, and the direct plot of log parasite density versus temperature were examined. The parasitaemia associated with an increase of 1°C from 37.5°C was determined by calculating the value between any two temperatures and the difference in parasitaemia on the semi-log plots and the non-log-transformed parasitaemias. A multiple logistic regression model was used to test the association between hypeparasitaemia (yes or no at presentation) and factors that were significant at univariate analysis: age, duration of illness before presentation, and presence of fever (temperature > 37.4°C). Because the study was conducted over a period of 3 years, time in years since the commencement of study was included as a covariate in the model. P-values of < 0.05 were taken to indicate significant differences.

## Results

During the study period, 6807 children with illnesses suspected to be or diagnosed as malaria were evaluated. Parasitaemia was present in 3338 children, a parasite rate of 49%. The clinical characteristics of the 3338 children enrolled in the study are summarized in Table 1. Twenty eight percent of parasite-positive children were aged less than 5 years, and hyperparasitaemia occurred in 3%. Parasitaemia > 150,000/μl was found in 8% of the children (Table 2).

**Table 1:** Characteristics of children with parasitaemia at baseline

Parameter	Value
**No. of children**	3338
Female (%)	1591 (48)
Aged < 5 years	938
Febrile (> 37.4°C)	1701
Hyperparasitaemic (> 250,000 (/μl of blood)	97
Anemic (< 30%)	461 (n = 1794)*
(< 15%)	8**
**Mean value (range) for**:	
Age (year)	7.4 (0.16 - 17)
Weight (kg)	21.7 (5 - 71)
Duration of illness (d)	3.2 (1 - 21)
Temperature (°C)	37.7 (35 - 41.1)
Hematocrit (%) GMPD (/μl of blood)	32.2 (10 - 51) 9440 (750 - 2,250,000)

*24 of 70 children with hyperparasitaemia and 437 of 1724 children without hyperparasitaemia had a PCV < 30% (P = 0.09).

** 1 of 70 children with hyperparasitaemia and 7 of 1724 children without hyperparasitaemia had a PCV < 15% (P = 0.27).

**Table 2:** Distribution of parasitaemia in children enrolled in the study

Period (year)	No. screened	No. positive for P. falciparum	Parasite density (/μl of blood)		
			
			> 250,000	> 150,000 - 250,000	< 150,000
2008	1956	912	11	39	862
2009	2853	1469	50	77	1342
2010	1998	957	36	56	865

### Relationship between body temperature, fever and parasitaemia

**Figure 1:**
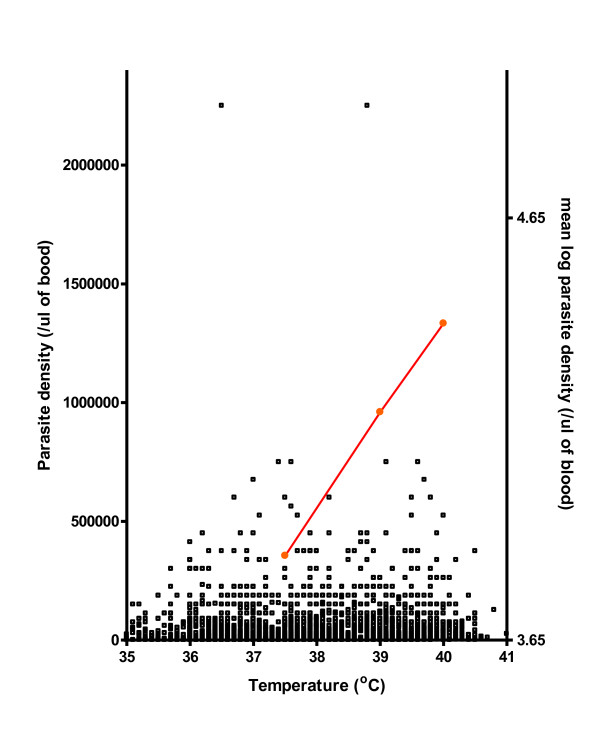
**Relationship between parasite density and body temperature and semi-log plot of mean parasitaemia (at a particular temperature) versus temperature**.

There was a significant positive correlation between body temperature and parasitaemia (r = 0.26, P < 0.000001, n = 3175). There was also a significant relationship between log parasitaemia and body temperature (Figure 1) such that an increase in parasitaemia of 350,000/μl resulted in a 1°C increase in body temperature above 37.5°C.

### Factors associated with hyperparasitaemia at presentation

Factors associated with hyperparasitaemia at enrolment are presented in Table 3. An age < 11 years, duration of illness ≤ 3 d, presence of fever, and enrolment after year 2008 were related to the presence of hyperparasitaemia at enrolment. Neither packed cell volume at presentation nor season of presentation was related to the presence of hyperparasitaemia (Table 3).

**Table 3:** Univariable analysis of potential risk factors for hyperparasitemia in children with *Plasmodium falciparum *malaria

Parameter	Number of children		Crude OR (95% CI)	P value
			
	Screened	HP		
Age (year)				
> 11	645	6	1	0.0001
≤ 11	2693	91	3.72 (1.62-8.54)	
Gender				
Male	1729	52	1	0.83
Female	1591	45	1.06 (0.71 - 1.59)	
Duration (d)				
> 3	677	10	1	0.013
≤ 3	2406	78	2.23 (1.15 - 4.34)	
Temp (°C)				
< 37.5	1474	25	1	0.001
≥ 37.5	1701	63	2.22 (1.39 - 3.56)	
Haematocrit (%)				
< 30	461	24	1	0.09
≥ 30	1333	46	1.53 (0.92 - 2.54)	
Year of enrolment				
during 2008	912	11	1	0.0001
After 2008	2426	86	3.01 (1.60 - 5.66)	
Season*				
Wet	2540	79	1	0.22
Dry	798	18	1.39 (0.82 - 2.33)	

*Wet season, April to October; dry season, November to March

### Risk factors for hyperparasitaemia at presentation

In the multivariable analysis, an age < 11 years, presence of fever, and enrolment after year 2008 were found to be independent risk factors for the presence of hyperparasitaemia at enrolment (Table 4).

**Table 4:** Multivariable analysis of independent risk factors for hyperparasitaemia in children with *Plasmodium falciparum *malaria

Parameter	**Total No**.	No. with HP	Crude OR (95% CI)	P value	Adjusted OR (95% CI)	P value
Age (year)						
> 11	645	6	1	0.0001	1	0.014
≤ 11	2693	91	3.72 (1.62-8.54)		2.85 (1.23-6.61)	
Duration (d)						
> 3	677	10	1	0.013	1	0.07
≤ 3	2406	78	2.23 (1.15 - 4.34)		1.85 (0.94 - 3.63)	
Temp (°C)						
< 37.5	1474	25	1	0.001	1	0.005
≥ 37.5	1701	63	2.22 (1.39 - 3.56)		2.02 (1.23 - 3.29)	
Year of enrolment						
during 2008	912	11	1	0.0001	1	0.001
After 2008	2426	86	3.01 (1.60 - 5.66)		3.07 (1.57 - 5.99)	

HP, hyperparasitaemia (asexual parasites > 250,000/μl of blood)

### Risk for progression to other forms of severe malaria

Eleven of 3338 children (0.3%) progressed to cerebral malaria: 7 of these had hyperparasitaemia and 4 had no hyperparasitaemia. The difference between the two proportions was significant (P < 0.0001, by Fisher exact test). Seven of the children were < 5 year olds. The risk of progression was considerably higher in < 5 year olds with hyperparasitaemia (5 of 30 *v *2 of 67 P = 0.02). One of the 3338 children died from cerebral malaria; the child had no hyperparasitaemia at presentation.

## Discussion

Although malaria is major cause of childhood morbidity and mortality in sub-Saharan Africa and hyperparasitaemia is considered a feature of severe malaria [1,2], few studies from this region have described the risk factors for hyperparasitaemia in this population. In the current study, there was clearly a broader age range for susceptibility to hyperparasitaemia-a departure from those of other forms of severe malaria in African children, for example, cerebral malaria, where children < 5 year olds are generally more susceptible.

As was expected, duration of illness < 4 days was associated with increased risk of hyperparasitaemia. This relatively short duration is reminiscent of the short duration of illness for other forms of severe malaria, for example, cerebral malaria where a short duration of illness is the norm [1]. However a short duration of illness was not an independent risk factor for hyperparasitaemia.

For two amongst many other reasons, it was surprising that anemia in under five year-olds, considered a surrogate marker of malaria disease burden in African children [22], was not associated with hyperparasitaemia in the cohort of children evaluated: a parasitaemia > 10,000/μl is a risk factor for malarial anaemia in children from this endemic area [23]; malaria is a major cause of anaemia in African children [24,25]. However, anemia was present in over 25% of cohort of children evaluated-prevalence considerably less than 39% in young children from the same endemic area [23]. The lack of association between anaemia and hyperparasitaemia may be due to the fact that anaemia may be multi-factorial in African children and the contribution of haemoglobinopathies and malnutrition to the anemia in these children may be considerable [26-28].

In a study from rural areas of Nigeria, Fasan & Lambo (1969)[29], and Delfini (1973)[30] found that increases in body temperature were associated with increasing parasitaemia in children with malaria but the relationship between density and temperature in the individual patients was not explored. In the present study, there was a significant relationship between log parasitaemia and body temperature such that an increase in parasitaemia of 350,000/μl resulted in a 1°C increase in body temperature above 37.5°C. This represents a substantial increase in asexual parasitaemia per unit rise in body temperature in this cohort of children. Additionally, fever was an independent risk factor for hyperparasitaemia. Indeed, children with fever were twice as likely to have a hyperparasitaemia compared to children without fever. This relationship and the associated risk may have been partially attenuated by the prior administration of antipyretic agents before presentation because approximately 68% of parents or guardians in this endemic area would give the antipyretic, acetaminophen, to their children at some point during a febrile illness before consultation (Sowunmi, unpublished).

In contradistinction to the finding in an area of lesser intensity of transmission, for example, on the Thai-Burmese border, where hyperparasitaemia is associated with the dry season [31], there was no association between season and the risk of hyperparasitaemia in this area of intense transmission. The reasons for the lack of association in the present study are unclear but more studies are required. It is also intriguing that enrolment after 2008 was an independent risk factor for hyperparasitaemia. There is, as yet, no ready explanation for this observation.

All the possible risk factors for hyperparasitaemia were not captured in the present study. Recent histories of diarrhoea and the presence of gametocytes, a finding associated with hyperparasitaemia in Thailand [9], for examples, were not considered. Nevertheless, a large number of children were evaluated. Hyperparasitaemia was associated with increased risk of progression to cerebral malaria suggesting that hyperparasitaemia should not be considered as variabe depending on epidemiology setting in childhood malaria as was done in the original WHO document [1] but be retained as a component of severe malaria at all times in children < 5 years. Thus, the results described in this study population should have significance in shaping the diagnosis of and guidelines for the management of hyperparasitaemia in children in African setting.

## Conclusion

In conclusion, a relatively broader age range, fever and enrolment after year 2008 are independent risk factors for hyperparasitaemia in malarious children in this endemic area. Hyperparasitaemia is significantly associated with an increased risk of progression to cerebral malaria in young children < 5 years old.

## Conflict of interests

The authors declare that they have no competing interests.

## Authors' contributions

AS led the design, conduct, data analysis and manuscript preparation, TMO was involved in conduct, data analysis and manuscript preparation, GOG and CTH were involved in design, conduct and preparation of the manuscript. All authors read and approved the final version of the manuscript.

## Pre-publication history

The pre-publication history for this paper can be accessed here:

http://www.biomedcentral.com/1471-2334/11/268/prepub
